# Supplementary material for: The Suppressive Effects of Biochar on Above- and Belowground Plant Pathogens and Pests: A Review
Source: Plants (Basel). 2022 Nov 17;11(22):3144. doi: 10.3390/plants11223144 (PMC9695804; doi:10.3390/plants11223144)
Supplement: Supplementary file 1 [file plants-11-03144-s001.zip › plants-1983920-supplementary.pdf]

Review

Supplementary Materials: The Suppressive Effects of Biochar on Above- and Belowground Plant Pathogens and Pests: A Review

Giuseppina Iacomino [1,*], Mohamed Idbella [1], Stefania Laudonia [1,2], Francesco Vinale [2,3] and Giuliano Bonanomi [1,4]

[1] Department of Agricultural Sciences, University of Naples Federico II, 80055 Portici, Italy
[2] Center for Studies on Bioinspired Agro-Environmental Technology, BAT Center, University of Naples Federico II, 80055 Portici, Italy
[3] Department of Veterinary Medicine and Animal Productions, University of Naples Federico II, 80137 Naples, Italy
[4] Task Force on Microbiome Studies, University of Naples Federico II, 80055 Portici, Italy
* Correspondence: giuseppina.iacomino@unina.it

Table S1. Count of study cases reporting different plants hosting the corresponding pathogens.

Host Plant	N° of case studies
Cucumis sativus	3
Morus alba	3
Asparagus officinalis	3
Glycine max	4
Lactuca sativa	5
Solanum tuberosum	5
Capsicum annum	5
Panax notoginseng	7
Jatropha curcas	7
Others	27
Solanum lycopersicum	49

Table S2. Count of study cases reporting different plant pathogens and pest species.

Pathogens & pests	N° of case studies
Parasitic plants	2
Viruses	3
Oomycetes	3
Nematodes	4
Insects	4
Bacteria	8

Fungi	28

Table S3. Count of study cases reporting different pathogen and pest species at different taxonomic level.

**Pathogen and pest**	**Species**	**N° of case studies**
Fungi	*Fusarium oxysporum* f. sp. *radicis lycopersici*	12
Fungi	*Rhizoctonia solani*	10
Fungi	*Botrytis cinerea*	5
Fungi	*Fusarium oxysporum*	5
Fungi	*Alternaria solani*	4
Fungi	*Fusarium solani*	4
Fungi	*Sclerotinia sclerotiorum*	4
Fungi	*Fusarium* spp.	3
Fungi	*Fusarium verticillioides*	2
Fungi	*Aspergillus niger*	2
Fungi	*Ilyonectria* spp.	1
Fungi	*Magnaporthe oryzae*	1
Fungi	*Ganoderma lucidum*	1
Fungi	*Penicillium italicum*	1
Fungi	*Trichoderma harzianum*	1
Fungi	*Verticillium dahliae*	1
Fungi	*Phyllactinia corylea*	1
Fungi	*Pseudocercospora mori*	1
Fungi	*Leveillula taurica*	1
Fungi	*Fusarium* f. sp. *asparagi*	1
Fungi	*Fusarium proliferatum*	1
Fungi	*Fusarium virguliforme*	1
Fungi	*Cylindrocarpon destructans*	1
Fungi	*Macrophomina phaseolina*	1
Fungi	*Stemphylium vesicarium*	1
Fungi	*Sclerotium cepivorum*	1
Fungi	*Sclerotium rolfsii*	1
Fungi	*Fusarium torulosum*	1
Nematode	*Meloidogyne incognita*	4
Nematode	*Globodera rostochiensis*	1
Nematode	*Globodera pallida*	1
Nematode	*Pratylenchus penetrans*	1
Virus	Tomato yellow leaf curl virus	3
Virus	Tomato mosaic virus	2

Oomycete	*Pythium aphanidermatum*	2
Oomycete	*Phytophthora infestans*	1
Oomycete	*Phytophthora capsici*	3
Bacteria	*Ralstonia solanacearum*	10
Bacteria	*Kosakonia sacchari*	1
Bacteria	*Agrobacterium tumefaciens*	1
Bacteria	*Pseudomonas syringae*	1
Bacteria	*Pseudomonas viridiflava*	1
Bacteria	*Bacillus subtilis*	1
Bacteria	*Lysobacter* sp.	1
Bacteria	*Streptomyces scabies*	1
Insect	*Maconellicoccus hirsutus*	1
Insect	*Epitrix fuscula*	2
Insect	*Cnaphalocrocis medinalis*	1
Insect	*Dorylus orientalis*	1
Plant holoparasite	*Phelipanche aegyptiaca*	1
Plant holoparasite	*Orobanche crenata*	1
